# Effects of Homocysteine Circulating Levels on Human Spontaneous Fertility and In Vitro Fertilization Outcomes: A Literature Review

**DOI:** 10.3390/nu17203211

**Published:** 2025-10-13

**Authors:** Alberto Revelli, Anna Maria Nuzzo, Laura Moretti, Silvana Arduino, Sofia Roero, Roberto Scali, Lorenzo Scali, Gianluca Gennarelli, Francesca Gigliotti, Marlisa Gatto, Alessandro Rolfo

**Affiliations:** 1 Department of Surgical Sciences, Gynecology and Obstetrics 2, City of Health and Science-S. Anna University Hospital, University of Turin, 10126 Turin, Italy; alberto.revelli@unito.it (A.R.); l.moretti@unito.it (L.M.); arduino.silvana70@gmail.com (S.A.); sofia.roero@unito.it (S.R.); scalir61@gmail.com (R.S.); lorenzo.scali@gmail.com (L.S.); francesca.gigliotti@icloud.com (F.G.); marlisa.gatto@unito.it (M.G.); alessandro.rolfo@unito.it (A.R.); 2 Obstetrics and Gynecology 1U, Physiopathology of Reproduction and IVF Unit, Department of Surgical Sciences, Sant Anna Hospital, University of Turin, 10126 Turin, Italy; ggennarelli@cittadellasalute.to.it

**Keywords:** homocysteine, hyperomocysteinemia, MTHFR, folic acid, micronutrients, human fertility, in vitro fertilization

## Abstract

**Background:** Homocysteine (Hcy) plays a pivotal role in human reproduction, influencing gamete quality, embryo development, implantation, and pregnancy outcomes. It is central to folate and methionine metabolism and supports methylation-dependent epigenetic processes. Hyperhomocysteinemia (HHcy) exerts diverse biological effects and is associated with reproductive impairments in both sexes, affecting both spontaneous fertility and the outcome of assisted reproduction, including In Vitro Fertilization (IVF). Although the mechanisms of HHcy toxicity in reproduction are not fully understood, significant progress has been made in elucidating its effects. The emerging picture is complex, as Hcy and its metabolites impact biomolecules and cellular processes in a tissue- and sex-specific manner. **Results**: In men, HHcy compromises sperm deoxyribonucleic acid (DNA) integrity, methylation, and testicular microcirculation, reducing fertility potential. In women, HHcy disrupts follicular growth, oocyte competence, embryo quality, and endometrial receptivity, increasing the risk of implantation failure, miscarriage, and pregnancy complications. In assisted reproduction, HHcy and 5,10-methylenetetrahydrofolate reductase (MTHFR) variants may lower oocyte yield and embryo quality, although folate and B-vitamin supplementation can mitigate these effects. **Conclusions**: These effects largely reflect oxidative, inflammatory, vascular and epigenetic mechanisms, highlighting Hcy as a modifiable factor for improving natural fertility, optimizing IVF outcomes, and supporting healthy offspring development.

## 1. Introduction

Large evidence shows that circulating micronutrients may affect several aspects of human reproduction, such as gamete production and competence, embryo development, implantation and placentation, as well as the subsequent pregnancy development and even the future health of the offspring [1].

Among micronutrients, the ammino acid homocysteine (Hcy) plays an important role in the maintenance of cell homeostasis: its metabolism is interconnected with that of folic acid, as well as with the synthesis and recycling of the essential ammino acid methionine (Met). Thus, disturbances in Hcy homeostasis can directly impact epigenetic regulation and methylation-dependent processes.

The excess of circulating Hcy, hyperhomocysteinemia (HHcy) has been associated with various human pathologies, including some forms of reproductive impairment in both sexes. In these cases, a better prognosis might be achieved by reducing Hcy levels via supplementation with appropriate micronutrients [2]. HHcy can arise from insufficient intake of B-vitamins (B9, B12, B6) and betaine (essential cofactors of Hcy-metabolizing enzymes), and/or from excessive methionine intake (the sole precursor of Hcy in the human body), and/or from mutations in genes encoding key enzymes such as cystathionine β-synthase (CβS), MTHFR and methionine synthase (MS) [3]. Although several molecular pathways underlying HHcy toxicity have been described [4,5,6], establishing causality between HHcy and associated diseases remains challenging. This is partly due to the heterogeneity of Hcy molecular forms [7,8], each one exerting distinct, and possibly sex-specific, effects on biological molecules [9], tissues, and organs [10,11]. Indeed, the pathophysiological consequences of HHcy likely represent the cumulative effects of Hcy and its metabolites, including homocysteine-thiolactone, *N*-Hcy-proteins, S-Hcy-proteins, S-Adenosylhomocysteine (SAH), and low-molecular-weight disulfides [10].

This review focuses on the complex effects of Hcy on human reproductive function, examining at a molecular level causes and consequences of HHcy on human fertility. Both spontaneous fecundity and the outcomes of the most advanced reproductive technologies, such as in vitro fertilization (IVF), will be examined, with a particular emphasis on epigenetic regulation and gamete/embryo competence.

## 2. Methodology

This review was designed to summarize current evidence on the circulating Hcy role in human reproduction, with particular emphasis on male and female fertility and outcomes of assisted reproduction, including IVF.

### 2.1. Literature Search Strategy

A comprehensive search of the literature was conducted in PubMed, Scopus, and Web of Science databases up to September 2025. The following keywords were used: homocysteine, hyperhomocysteinemia, MTHFR, folate, vitamin B12, fertility, male infertility, female infertility, spermatogenesis, oocyte competence, embryo quality, endometrial receptivity, implantation, pregnancy outcome, and in vitro fertilization. Reference lists of relevant reviews and original articles were also checked to identify additional publications.

### 2.2. Inclusion and Exclusion Criteria

We included original research articles (clinical studies, case–control studies, cohort studies, randomized controlled trials), systematic reviews, meta-analyses, and relevant experimental studies in animals that addressed mechanistic aspects of Hcy in reproduction. Only articles published in English were considered. Studies that did not focus on reproduction or did not provide data on circulating Hcy, folate metabolism, or reproductive outcomes were excluded.

### 2.3. Study Selection and Data Extraction

Two authors independently screened titles and abstracts to assess eligibility. Full texts of potentially relevant articles were retrieved and evaluated. Disagreements were resolved through discussion with a third reviewer. Data were extracted and narratively synthesized, focusing on Hcy metabolism and regulation, effects of HHcy on male fertility, effects of HHcy on female fertility, impact of Hcy levels and MTHFR polymorphisms on IVF outcomes.

### 2.4. Quality Assessment and Limitations

Given the narrative nature of this review, no formal meta-analysis or quantitative synthesis was performed. However, particular attention was paid to study design, sample size, presence of confounder adjustment (e.g., age, diet, BMI, smoking), and ethnic/geographical stratification, as these factors critically influence the association between Hcy metabolism and reproductive outcomes.

## 3. Metabolism of Homocysteine

The ammino acid Hcy cannot be found in dietary proteins, since it derives from the metabolism of another ammino acid, Met, which is contained in meat, eggs, and legumes. Met plays a crucial role in human metabolism, acting as methyl group donor, thus allowing the synthesis of methylated compounds. Furthermore, Met provides the carbon skeleton for polyamine synthesis, and Met-sulfate is fundamental for the synthesis of sulfur-containing ammino acids [12]. Hcy is central to the biochemical pathways that regulate Met availability within the body.

**Figure 1:**
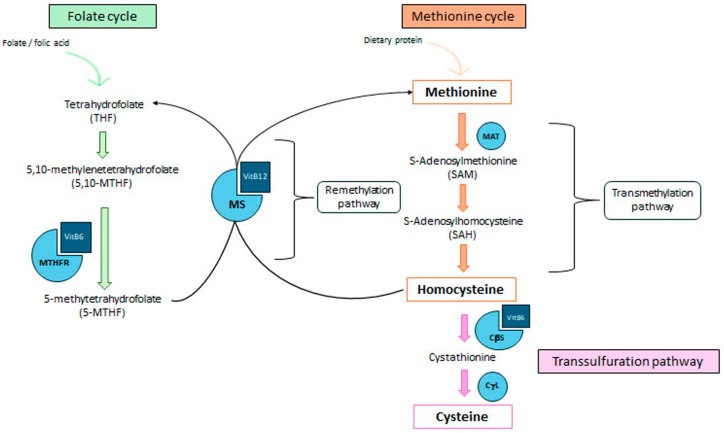
Schematic representation of Hcy metabolism. Green, folate cycle: remethylation of Hcy to methionine through folate- and vitamin B12–dependent reactions (THF—Tetrahydrofolate; 5,10-MTHF—5,10-methylenetetrahydrofolate; MTHFR—5,10-Methylenetetrahydrofolate reductase; 5-MTHF—5-methyltetrahydrofolate; MS—Methionine synthase); orange, methionine cycle: production of homocysteine from methionine (MAT—Methionine adenosyltransferase; SAM—S-adenosylmethionine; SAH—S-adenosylhomocysteine); pink, transsulfuration pathway: breakdown of homocysteine and production of cysteine (CβS—Cystathionine β-synthase; CγL—Cystathionine γ-lyase).

Figure 1 schematically shows that Hcy derives from the demethylation of Met, accomplished through a sequence of three steps, grouped under the name of “transmethylation pathway” [13].

In the first step, the enzyme met-adenosyl-tranferase transfers the adenosyl portion of adenosine triphosphate to Met, generating S-Adenosyl-Met (SAM). In the second step, SAM donates its methyl group to various acceptors (deoxyribonucleic acid (DNA), ribonucleic acid (RNA), proteins, etc.): this transfer is named “transmethylation”. The remaining compound SAH is then cleaved into Hcy plus adenosine by the enzyme adenosyl-Hcy-hydrolase. The overall effectiveness of transmethylation pathway is regulated by the intracellular availability of the intermediate substrates SAM and SAH.

After Hcy is formed, it is catabolized via two different pathways: approximately 70% of Hcy undergoes recycling into Met (“remethylation pathway”), whereas the remaining 30% is converted into the ammino acid Cysteine (Cys), following the “transsulfuration pathway” [13].

In the remethylation pathway, the enzyme MS, which uses vitamin B12 as cofactor, restores Met by transferring to Cys a methyl group taken from 5-Methyl-Tetraidrofolate (5-MTHF), a substance deriving from the Folate Cycle (Figure 1). The Folate Cycle starts from dietary folate or folic acid, a group B vitamin contained in almost all fruits and vegetables. The folate/folic acid acceptor inside the cell is Tetrahydrofolate (THF), in turn converted by the enzyme 5,10-Methylenetetrahydrofolate Reductase (MTHFR) into 5,10-methylene-THF, and then into 5-MTHF [13]. The synthesis of 5-MTHF depends on both the folate/folic acid dietary availability, and by the activity of MTHFR, that uses vitamin B6 as coenzyme. Finally, MTHFR removes a methyl group from 5-MTHF, which is transfered first to the vitamin B12 coenzyme, then to Hcy, re-forming Met (“remethylation”). Any impairment of MTHFR enzymatic function (e.g., caused by a mutation on the MTHFR gene), can reduce Hcy conversion into Met, resulting in the accumulation of Hcy and increase of circulating concentrations (HHcy). Vitamins B6 and B12 give a central contribution to Hcy catabolism as well, acting as enzyme cofactors. Whereas these vitamins are rather abundant in the diet, particularly in some foods of animal origin (e.g., meat, milk, eggs) and in wholegrain, their deficiency reduces the rigeneration of Met from Hcy, and causes HHcy.

The alternative catabolic route of Hcy is the “transsulfuration pathway”, which catabolizes Hcy by converting it into Cys. The enzyme CβS, that uses vitamin B6 as cofactor, transforms Hcy into cystathionine, in turn cleaved subsequently into Cys by cystathionine-γ-lyase [13]. The trans-sulfuration pathway starts to function as a “reinforcement pathway” when the circulating concentration of Hcy increases relevantly, e.g., after abundant protein (Met) intake during a meal [14].

In some specific tissues (liver and kidney), but not in the whole body, another alternative metabolic pathway contributing to Hcy catabolism exists: betaine acts as methyl group donor, through the activity of the enzyme betaine-Hcy-methyltransferase, and contributes to lower Hcy intracellular levels and regenerate Met. Betaine derives from the oxidation of choline, which comes mainly from diet, being contained in seafood, legumes, wheat germ, spinach [15].

The Hcy that is not catabolized enters blood circulation, where it can be found in three different forms: about 70–85% is bound to albumin, 25–30% is bound to soluble disulphides, and 1–3% is unbound. There is no general agreement about which circulating Hcy range should be considered normal; some changes are observed post-prandially, so the collection of fasting blood samples is recommended to check Hcy serum level. In the past, different cut-offs have been proposed as maximal normal values [16]: some authors considered 5–25 μmol/L as the ideal level for any age range [17], while others proposed values within 4.7–14.6 μmol/L and 18.8–49.7 μmol/L for fasting and 6 h post-protein/Met load, respectively [18]. Nowadays, most authors consider as normal a circulating Hcy level between 5 and 15 μmol/L; HHcy is considered mild when ranging from 15 to 30 μmol/L, intermediate between 30 and 100 μmol/L, serious when exceeding 100 μmol/L [19].

Circulating Hcy concentrations are reduced by estrogens, being lower in females, and during the follicular phase of the menstrual cycle, when estrogens predominate [19]. Furthermore, Hcy increases in post-menopausal women or after bilateral oophorectomy [20]. The effect of estrogens appears to be counteracted by progesterone derivates: estro-progestin hormonal replacement therapy showed no significant effect on Hcy levels [21], nor were they were affected by oral contraceptives containing estrogens and progestins [22]. Rather surprisingly, in a prospective study in IVF patients, Hcy concentration was not affected by gonadotropin-releasing hormone (GnRH)-agonists, known to induce a chronic, reversible hypoestrogenic state [23]. However, these patients usually are prescribed folate and B-group vitamin supplementation for weeks before starting the GnRH-agonist, which would easily explain the observed results, given the direct lowering effects of these vitamins on Hcy serum concentrations.

## 4. Hyperhomocysteinemia

Hyperhomocysteinemia is considered a reliable biomarker of chronic and acute inflammatory status, and has been associated with increased risk of atherosclerotic, thromboembolic and neurodegenerative disorders [2].

The excess Hcy is exported from the cells into the bloodstream, where it is partially inactivated via binding to albumin. Part of the metabolically active portion of circulating Hcy reacts with Cys residues to homocysteinylate them, while another part undergoes oxidation to form the disulfide Hcy, mixed disulfides or other low-molecular-weight thiols [24]. Homocysteinylated proteins are able to exert cytotoxic, pro-inflammatory, and prothrombotic activities [25].

Overall, HHcy can induce endothelial damage by increasing oxidative stress, reducing the activity of nitric oxide (NO), enhancing the proliferation of vascular smooth cells, and increasing the local synthesis of inflammatory cytokines [26]. It also causes visceral fat accumulation and weight increase [27]. In neurodegenerative diseases, Hcy neurotoxicity is due to altered methylation and/or increased calcium influx into neural cells [28], amyloid and tau protein accumulation [29] and neuronal apoptosis [30].

HHcy may be associated with several conditions, including hypothyroidism, renal dysfunction, voluptuary habits (cigarette smoking, high alcohol consumption, high caffeine intake, use of drugs), and lifestyle (limited physical exercise) [31]. However, from a pathogenetic standpoint, there are two main factors causing HHcy: (a) nutritional deficiency of compounds involved in Hcy catabolism (folate/folic acid, vitaminsB6 and B12, betaine), and (b) reduced function of enzymes involved in Hcy catabolism, in turn linked to mutations of the genes encoding for them.

### 4.1. Nutritional Defects

The dietary intake of folate/folic acid, vitamins B6 and B12, and proteins rich in Met is a major factor regulating Hcy circulating level [32]. Dietary folic acid is low, especially in areas where fresh fruit and vegetables are poorly consumed, or where artificial fortification of wholegrain products is rare or totally absent. Low serum concentrations of vitamins B6 and B12 are also associated with insufficient nutritional intake, a condition more frequent than generally believed in some geographic areas, in which approximately 40% of women aged 21–44 years have a diet with insufficient vitamin B-group content [33].

Micronutrient supplementation provides relevant benefits to Hcy metabolism; a reduction in plasma Hcy levels was demonstrated after diet supplementation with folic acid, vitamins B6 and B12 [34]. The effect of nutritional supplementation appeared to be synergic: 0.5–5 mg/day folic acid supplementation lowered serum Hcy by 25%, whereas the co-administration of 0.4 mg/day vitamin B12 further reduced Hcy levels by 7%; furthermore, vitamin B6 intake was able to reduce Hcy plasma levels even after Met loading [34]. A metanalysis showed that the daily supplementation with 0.5–5 mg folic acid plus 0.5 mg vitamin B12 was able to reduce plasma Hcy concentration by approximately 30% [35].

Folic acid is the form contained in foods and in several nutritional supplements available worldwide: inside the body, it isconverted into the more bioactive derivative 5-MTHF [36,37]. Nowadays, 5-MTHF is also available for exogenous administration and is incorporated by all cells expressing folate-receptor-α, e.g., those in the proximal tubules of kidney, whereas the two other folate-receptors (β and γ) show lower affinity for it; the membrane carrier that brings 5-MTHF into the cell is ubiquitous [38] and 5-MHTF is a valid alternative to folic acid for nutritional integration.

### 4.2. Defective Function of Enzymes Involved in Hcy Catabolism

The most important enzymes regulating Hcy levels are encoded by genes that show various single nucleotide olymorphisms (SNPs) affecting their activity [39,40,41].

The enzyme most frequently involved as a cause of HHcy is MTHFR (Figure 1), whose gene (formed by 33 exons) is located at the end of the short arm of chromosome 1 [42,43]. There are approximately 20 SNPs of MTHFR gene, most of them reducing the enzyme activity; the two best known MTHFR polymorphisms are 677C>T (alanine at position 222 replaced by valine) and 1298A>C (glutamate at position 249 replaced by alanine) [44]. Both polymorphisms reduce enzyme activity. Compared with the 677CC wild-type genotype, the 677C>T variant has 60% function, whereas the 677TT variant has only 10–20% of enzyme activity due to a markedly decreased affinity for vitamin B6 cofactor [45]. Approximately 10% of the world population has the MTHFR 677TT genotype; however, the frequency of this omozygotic polymorphism rises to 25% in certain geographic areas (e.g., southern Italy). The MTHFR 677TT genotype is able to alter Hcy metabolism in particular when folate availability is low [46], suggesting a genetic-diet interaction in determining the final effect [47].

Other enzymes that play a relevant role in the regulation of Hcy catabolism are MS, whose polymorphisms MS 2756A>G reduces the activity of the remethylation cycle [47] and cystathionine-β-synthase, whose polymorphism 833T>C, significantly decreases the activity of thetrans-sulfuration pathway [48].

## 5. Homocysteine and Male Fertility

The folate- and vitamin B12-dependent remethylation of Hcy to Met is essential for a highly proliferating epithelium like the one paving the seminiferous tubules of the testicles. Met is extremely important for human spermatogenesis since it provides methyl groups used to methylate sperm DNA [49].

If Hcy blood levels increase over the normal threshold, HHcy causes abnormal sperm DNA methylation, inducing oxidative stress and sperm DNA fragmentation, thus reducing fertility potential [50]. Furthermore, HHcy may provoke damage to the seminiferous tubules, by reducing blood flow in the testicular microcirculation [51]. Additional evidence supports a direct epigenetic contribution to male infertility. Studies investigating disrupted spermatogenesis used bisulphite genomic sequencing to analyze methylation profiles in spermatozoa DNA. These showed that abnormal spermatogenesis is associated with an increase in defective *H19* methylation, suggesting an association between abnormal genomic imprinting and hypospermatogenesis. Importantly, spermatozoa from oligozoospermic patients were found to carry an increased risk of transmitting imprinting errors [52]. Furthermore, experimental models have highlighted a paternal influence on embryo development: paternal exposure to hypomethylating agents such as 5-aza-2′-deoxycytidine interfered with normal male germ cell development, leading to a dose-dependent decline in global sperm DNA methylation (assessed by TLC) and increased preimplantation loss in progeny [50].

### 5.1. Dietary Intake/Supplementation of Folates and Vitamins

A large body of evidence suggests that a normal intake of folic acid and B-group vitamins is fundamental for the spermatogenetic epithelium [53]. High affinity folate-binding sites are found in human testicular tissue [54], whereas reduced serum folate concentrations have been observed in men with idiopatic infertility, in spite of normal semen parameters [55]. The seminal plasma, deriving from seminal vesicles and prostate, contains a folate-binding protein of epididymal origin [56] that promotes folate uptake by spermatozoa. The concentration of folate in seminal plasma, which is 1.5 times higher than in the blood, correlates negatively with the amount of reactive oxygen species [57] and with the sperm DNA fragmentation index, suggesting that folates in seminal plasma exert a protective role against local inflammation and sperm DNA damage [58].

Folate deprivation has been investigated in animal models. In rats, both a folic acid-deficient diet and the administration of folate metabolism inhibitors cause a significant reduction in sperm quality [59,60]. In MTHFR knock-out mice, whose MTHFR activity is totally abolished, seminiferous tubules appeared either devoid of spermatogenetic cells, or with a maturation block, associated with increased apoptosis [61].

In humans, folate deficiency or its altered metabolism, in the presence of HHcy, was observed to induce a defective methylation of H19 gene causing severe oligozoospermia [52] and to affect Fas ligand-induced apoptosis in the testicle, linked to impaired spermatogenesis [62]. Additionally, HHcy reduced testicular blood flow via damage to microcirculation within the gonad [51]. The negative effect of HHcy was also elicited at various levels of the male genital tract, via either enhancement of the local synthesis of inflammatory cytokines [63] or reduction of NO, known to regulate sperm motility, acrosome reaction and fertilization [64,65,66].

A few studies reported the effect of folate supplementation on human male fertility. The daily administration of 15 mg/d folic acid for 3 months increased sperm concentration and motility, with a beneficial effect on both sperm quality and local flogosis [67]. A randomized, placebo-controlled study on oligozoospermic men showed that folic acid supplementation (5 mg/day for 6 months) increased sperm density by 40%. However, when zinc was also added, a surprising 74% increase was obtained, showing a synergistic effect of the two nutritional elements [68]. Interestingly enough, the effect of folate plus zinc supplementation was limited to MTHFR wild-type 677CC carriers, whereas it was not observed in 677C>T heterozygotes and in homozygous TT mutants, whose residual MTHFR activity was probably insufficient to produce the beneficial effect [69]. Of note, the positive effect of the combined folic acid and zinc supplementation on subfertile patients was confirmed in another study by the same group [70].

### 5.2. Genetic Polymorphisms of Enzymes Involved in Met and Folate Cycles

#### 5.2.1. MTHFR 677C>T Polymorphism

The association between the MTHFR 667C>T polymorphism and male infertility was first investigated in 2001 [71]. Since then, many case–control studies have been performed, overall reaching inconsistent conclusions. In order to increase statistical power and the level of evidence, some meta-analyses have been published in recent years [47,72,73,74,75,76,77,78]. The included studies were performed in various ethnic groups, mainly Asian populations [77,78] or a mix of Asians and Caucasians [47,72,73,74,75]. Unfortunately, there was an overall relevant heterogeneity among studies. As an example, some studies compared infertile vs. fertile men, whereas others compared normozoospermic vs. dyspermic subjects. The two comparisons are indeed similar, but not fully equivalent, as a man belonging to an infertile couple can be normozoospermic, while a man with a mild sperm defect can belong to a fertile couple. Furthermore, several studies were not adjusted for confounding factors such as age, alcohol intake, smoking habit, etc. These potentials sources of bias were obviously imported into the metanalyses, contributing to the heterogeinity and reducing the overall quality of evidence.

In order to overcome these limitations a recent, large metanalysis applied strict quality assessment of the 59 studies included (11,767 infertile men and 10,591 controls) [79]. The metanalysis concluded that the MTHFR 677C>T polymorphism is likely to be associated with oligoasthenoteratozoospermia and male infertility. A further quite recent metanalysis, including 46 case–control studies with a total of 20,639 participants [80], reported a similar association, which was, however, stronger in Asian men than in caucasians.

Indeed, the notion of some relevant difference among ethnic groups is not new. Early studies on Indian patients with severe oligozoospermia or azoospermia [81] and on Korean [82] or Brasilian [83,84] infertile men, reported that carriers of mutant MTHFR polymorphism 677TT and 677C>T were more frequent among dyspermic patients than in fertile controls. A large study on a Chinese population showed that 677TT genetic variant was significantly associated with non-obstructive azoospermia and male infertility [85]. Several metanalyses [46,71,72,73,74,76,77,84] showed that the MTHFR variant 677C>T was associated with male infertility and semen alterations mainly in Asians. Of note, this observation has not been confirmed in Dutch [69] and Italian patients [86].

However, an interesting metanalysis by Ullah et al., indicated that the MTHFR 677C>T polymorphism was associated with an increased risk of male infertility also in some Caucasian populations, particularly those living in countries with lower income [87].

Thus, the ethnic differences emerging from several studies could possibly be explained by the higher presence of folic acid in the diet of Caucasians and in the higher nutritional vitamin intake of Europeans compared to Asians. The existence of a relevant interaction between nutritional status and genotype was clearly evidenced, in another context, in Spain, where the prevalence of MTHFR 677-mutated subjects doubled after the introduction of folic acid supplementation program for pregnant women. It is speculated that folic acid supplementation could have saved from miscarriage mutated fetuses [88]. Indeed, the presence of 3–4 mutated MTHFR alleles could be detected in aborted fetuses, but not in living neonates [89]. To further corroborate this hypothesis, a progressive increase of MTHFR genotypes with two mutated alleles was reported in younger subjects when compared with older ones, reflecting a folate supplementation-induced effect of genetic selection [90]. Interestingly enough, a study reported a significantly higher frequency of 677C>T or 677TT subjects among severe oligospermic and azoospermic men, but an increased vitamin B12 intake was effective in improving semen parameters [91]. Furthermore, a prospective study from North America showed that the MTHFR 677TT homozygous variant was associated with male infertility, but not in a subgroup of patients with a common deletion of Glutathione S-Transferase Mu 1 (GSTM1), a gene encoding glutathione transferase [92]. The GSTM1 deletion causes an increase in glutathione, which in turn stimulates SAM activity, thus counteracting the effects of an unfavorable MTHFR polymorphism. This evidence supports the notion that complex gene interactions should be considered when interpreting the results of studies on MTHFR polymorphisms.

Overall, the available results suggest that it could be worth including the evaluation of MTHFR polymorphism in male fertility diagnostic workout. Men with MTHFR 677C>T ot TT polymorphism could be given appropriate folic acid and vitamin supplementation in order to improve their sperm quality and fertility.

#### 5.2.2. MTHFR 1298A>C Polymorphism

The association between MTHFR 1298A>C polymorphism and male infertility risk was first investigated in 2005 [82]. In the last two decades, several studies and meta-analyses have been published on the topic [47,72,75,78,87,93,94,95,96]. Among them, one [87] considered exclusively caucasians, two only Asians [78,96], and seven both ethnic groups [47,72,75,94,95,97]. Overall, the results of these studies were inconsistent: some of them indicated that the MTHFR 1298A>C polymorphism was associated with an increased risk of male infertility [72,87,94,96,97], other found that it was not [47,75,78,95].

In the two more recent, larger metanalysis including 28 studies (5976 infertile males and 5774 controls) [79] and 46 studies (20,639 participants) [80], respectively, no significant association was observed between the MTHFR 1298A>C polymorphism and male fertility, neither considering fertile vs. infertile men, nor normospermic vs. dyspermic subjects.

## 6. Homocysteine and Female Fertility

Studies investigating the effect of HHcy on human spontaneous fertility are scarce, and knowledge in the field is so far limited. HHcy linked to the presence of unfavorable polymorphisms of MTHFR gene was reported to be significantly more frequent in women with unexplained infertility than in age and Body Mass Index (BMI)-matched fertile controls [96,97], although the underlying mechanism remains unclear.

The detrimental effect of HHcy on female fertility could be exerted at different levels. During follicular growth, HHcy-activated alterations of DNA methylation might impair the proliferation of granulosa cells, elicit a pro-apoptotic effect, and negatively affect follicular maturation [98]. Supporting this hypothesis, animal models showed that clinically relevant reductions in B vitamins and methionine status around conception led to widespread epigenetic modifications of DNA methylation in the fetal liver, as demonstrated by analysis of 1400 CpG sites using Restriction Landmark Genome Scanning (RLGS). These findings reinforce the concept that maternal one-carbon metabolism strongly influences epigenetic programming and developmental outcomes [99].

An increased level of oxidative stress and apoptosis is known to play a negative role in physiological events such as follicular maturation and cyclic endometrial regeneration, but also in specific pathologies affecting fertility and known to have a chronic inflammatory background, like pelvic endometriosis [100]. Indeed, HHcy was found to induce monocyte chemotactic protein-1 and interleukin-8 overproduction, in turn associated with the flogistic status at the basis of endometriosis-related infertility [101,102]. The induction of local inflammatory cytokine production and/or of local alteration of NO production, resulting in a overall increase of oxidative stress, is another possibility for HHcy to interfere with female fertility within the female genital tract, at the uterine or tubal level, acting both on sperm migration toward the egg, and on the biochemical microenvironment that supports fertilization and early embryo development within the salpinx [103].

Some studies, but not others [104], observed the presence of increased serum Hcy level in women with Polycystic Ovary Syndrome (PCOS) [105,106], in which HHcy was associated with higher BMI and reduced spontaneous ovulation rate [107]. A significant correlation between circulating Hcy levels and insulin resistance or hyperandrogenism, endocrine features associated with overweight/obesity and chronic anovulation, was reported by several studies [108,109,110,111,112,113,114,115,116] but not by others [117,118,119]. In the study of Badawy et al., HHcy was found in 41.1% of PCOS patients, with higher prevalence (47%) in those with insulin resistance than in those without (23%) [120]. The molecular mechanism underlying the Hcy effect over insulin resistance was identified in the cysteine-homocysteinilation of the proinsulin receptor in the endoplasmic reticulum [121]. As for Hcy/androgens interplay, HHcy was found to promote hyperandrogenism in women with PCOS, contributing to dyslipidemia [122], while dyhydrotestosterone, in turn, was observed to induce HHcy by reducing the activity of Hcy-catabolising enzymes [123].

Interestingly enough, the administration of metformin, frequently recommended in PCOS patients in order to reduce insulin resistance and promote the return of spontaneous ovulation, may further increase Hcy via induction of folate and vitamin B12 depletion [93,124,125]; the concomitant folate and B group vitamin supplementation may effectively avoid this undesired pharmacologic effect [93,125,126].

## 7. Hcy and In Vitro Fertilization (IVF)

The scenario of IVF is a very complex one, in which both the substrate (gametes and endometrium), the technical methodology, and a large series of details contribute to determine success rates. In this contest, Hcy metabolism might play a relevant role, given its strict correlation with DNA methylation, which is responsible for the epigenetic regulation of embryos in their first days of development. Indeed, the embryonic epigenetic programming via the methylation–demethylation cycle, is a central mechanism in the maintenance of genomic stability, not just during early embryo growth, but also later, during pregnancy and life after birth. The folate cycle, the methionine cycle, and the transsulfuration pathway are of pivotal importance for obtaining proper DNA methylation, essential for the appropriate gene regulation and expression in the offspring [127]. There is concern that IVF procedures may compromise normal imprinting and methylation. An increase in imprinting defects (e.g., Angelman’s and Beckwith-Wiedemann’s syndromes) has been reported following ART, possibly caused by loss of methylation. Aberrant methylation patterns at the two-cell stage may also serve as an indicator of early developmental failure [128]. Patients with MTHFR polymorphism 677C>T or 677TT undergoing IVF obtained a lower oocyte yield on average, after hormonal ovarian stimulation, and required a significantly higher follicle-stimulating hormone (FSH) dose than controls, suggesting a lower follicular responsiveness to gonadotropins [129]. Noticeably, the effect was observed to be more marked in “reproductively older” women (>35 years), suggesting a progressive impact of MTHFR polymorphism on the ovarian reserve and/or the ovarian responsiveness to hormones [103]. A recent study on infertile women confirmed that those with low ovarian reserve had on average higher circulating Hcy levels, and significantly higher incidence of HHcy [130]. Also, the quality of oocytes might be affected by Hcy circulating levels. Higher serum Hcy concentrations were found to be associated with a higher proportion of embryos with aneuploidy, that in most cases derive from aneuploid oocytes [131]. The existence of a negative impact of the MTHFR 677C>T polymorphism on the ovarian responsiveness to FSH could also explain the lower prevalence of the MTHFR 677T allele in women who spontaneously conceived dichorionic twins [132]; the observation suggests that HHcy could inhibit bifollicular ovulation, which is needed for spontaneous dichorionic conception or, alternatively, could predispose to the the “vanishing twin”, with precocious miscarriage of one of the twins.

Within the developing follicle, the biochemical environment in the follicular fluid (FF) deeply affects the acquisition of proper competence by the oocyte [133]. The latter, in turn, influences the growth of the embryo in the first 72 h post-fertilization. A low follicular fluid Hcy level was found to positively correlate with oocyte maturation and competence, with better embryos deriving from follicles whose fluid had low Hcy levels [134,135,136]. Accordingly, a negative correlation between follicular fluid Hcy concentration and the embryo quality on day 3 after IVF was reported [70]. FF Hcy also showed a negative correlation with clinical pregnancy after IVF; folate supplementation for two months prior to IVF reduced follicular fluid Hcy and increased the embryo implantation rate [137]. Indeed, folate and Hcy concentrations in follicular fluid are similar to those in blood [138] and may be reduced by folate supplementation [58,139]. In this regard, higher circulating Hcy levels were found to be associated with a low fertilization rate, and folate supplementation significantly improved the fertilization outcome [140].

The epigenetic regulation within the in vitro-produced embryo, linked to DNA methylation, may lead to chromatin remodeling and gene expression that affect the overall embryonic development. Soon after fertilization, the zygotic genome undergoes rapid demethylation differently, across implantation the embryonic ectoderm and mesoderm genome becomes hypermethylated, whereas the trophoblast remains hypomethylated. In mice, these processes might be enhanced by methyl donors provided by the folate-Met pathway, whose activity affects all further embryo development [141]. In sheep, the periconceptional supply of folate, vitamin B12 and methionine was observed to affect epigenetic methylation and phenotype in the conceptus [99]. Interestingly enough, Hcy levels in spent culture media of embryos at the cleavage stage were found to be negatively associated with the likelihood of pregnancy after the transfer in utero, suggesting the idea of adopting Hcy levels in the culture medium as a biomarker of embryo quality [142].

Considering the IVF overall success rate, some studies reported that the pregnancy rate obtained by the procedure was not dependent on the folate status or the MTHFR gene polymorphism [143,144,145,146]. A rather large study on 692 women undergoing IVF showed that MTHFR 677C>T polymorphism was not associated with the pregnancy rate [147]. Similarly, in other studies it was observed that both the genotypes MTHFR 677C>T and 1298AA were not able to influence the chance to have a viable pregnancy [148,149]. It has to be noted, however, that women undergoing IVF usually take prolonged folate supplementation before the procedure, and this may affect their Hcy circulating concentrations, eventually compensating for an unfavorable MTHFR polymorphism [150]. Moreover, obtaining a clinical pregnancy after IVF is something influenced by many different factors: studies applying multivariate analysis could possibly be the only way to understand the impact of circulationg Hcy on IVF outcome.

A recent retrospective analysis of women who underwent repeated unsuccessful embryo transfer found that serum levels of Hcy were related with both the number of failed transfers and the total number of transferred embryos, even when Hcy was within the normal range [151]. Also, other studies, but not all [152], reported a significantly higher prevalence of HHcy among women with embryo implantation failure [37,153,154]. Indeed, embryo implantation is a quite complex phenomenon, and Repeated Implantation Failure (RIF) is still a “black box” in which no explanation may be found in most cases: the research on a possible cause–effect relationship between Hcy metabolism and RIF could open novel, interesting perspectives.

## 8. Conclusions and Future Perspective

HHcy exerts multifaceted effects on human reproduction, influencing both male and female fertility through vascular, oxidative, inflammatory, and epigenetic mechanisms. In men, HHcy compromises sperm DNA integrity, epigenetic regulation, and testicular microcirculation, ultimately reducing fertility potential. The association between MTHFR 677C>T polymorphism and male infertility varies across populations: positive associations were reported in cohorts from Germany, South Korea, India, Italy, China, Brazil, and Iran, whereas no effect was observed in Dutch, French, Romanian, or South Indian populations [149,155]. The MTHFR 1298A>C polymorphism showed no overall effect, but subgroup analyses indicate increased risk in Asian populations. Dietary folate strongly modulates these genetic risks, as low seminal folate correlates with DNA damage, while supplementation with folinic acid or betaine mitigates adverse effects [148,149,150]. In women, elevated Hcy levels disrupt follicular growth, oocyte competence, embryo quality, and endometrial receptivity, increasing the risk of implantation failure, miscarriage, and adverse pregnancy outcomes. HHcy prevalence is particularly high in metabolic disorders such as PCOS, with geographic and ethnic variability; insulin resistance (frequently observed among overweight PCOS women) modulates Hcy metabolism only in women without thermolabile MTHFR polymorphism [104,105,149]. Follicular fluid Hcy predicts oocyte and embryo quality, being elevated in both PCOS and endometriosis, while androgens exacerbate Hcy accumulation and granulosa cell autophagy via mTOR inhibition [151]. Pharmacological factors, notably Metformin, further increase Hcy while lowering B12 and folate, effects counteracted by co-supplementation [137]. In assisted reproductive technologies, including IVF, HHcy and genetic variants such as MTHFR polymorphisms may lower oocyte yield and embryo quality, effects partially counteracted by adequate folate and B-vitamin supplementation. Genotype-specific folic acid supplementation (up to 800 μg/day for 677TT women in a Chinese cohort) neutralized the association between MTHFR variants and adverse outcomes. Elevated folate status also increases the probability of twin births after multiple embryo transfer, while high plasma Hcy is associated with increased miscarriage risk. Genotype-specific folic acid dosing (up to 800 μg/day for 677TT women in a Chinese cohort) neutralizes genetic risks [147]. Emerging evidence highlights the critical role of Hcy in epigenetic regulation, including DNA methylation, histone modifications, and non-coding RNA pathways, suggesting that HHcy may have long-term consequences on offspring health. Importantly, the influence of HHcy on reproductive outcomes is critically modulated by nutritional and lifestyle factors. Adequate intake of folate, B vitamins (B2, B6, B12), and methionine-rich foods directly affects Hcy metabolism, with deficiencies promoting HHcy and compromising gamete quality, DNA methylation, and embryo development. Similarly, supplementation practices can mask or modify the effects of MTHFR polymorphisms, as evidenced by studies showing that genotype-specific folic acid or active folate (5-MTHF) intake neutralizes genetic risk and improves IVF outcomes [127,137,140,143,150]. Lifestyle behaviors, including smoking and alcohol consumption, further exacerbate oxidative stress and elevate Hcy, impairing oocyte and sperm competence [104,130,140]. Metabolic factors such as insulin resistance and pharmacological exposures, notably Metformin, can also influence Hcy levels and reproductive success, highlighting the importance of co-supplementation and careful control of confounding variables.

Future research should elucidate the tissue- and sex-specific molecular pathways linking HHcy to gamete and embryo competence, implantation, and early development. Monitoring circulating and follicular fluid Hcy through prospective longitudinal studies, along with epigenetic signatures using mendelian randomization approaches, could improve the prediction of fertility potential and IVF outcomes. Given these established links and existing methodology (e.g., bisulphite genomic sequencing or RLGS), the inclusion of epigenetic assays in gametes and embryos serves as a vital control to directly assess the role of DNA methylation changes, potentially providing a mechanistic explanation for observed reproductive outcomes, such as fertilization rates, embryo quality, or implantation failure. Intervention trials evaluating the effects of targeted micronutrient supplementation (e.g., folate, vitamins B6/B12, 5-MTHF) on HHcy reduction and reproductive endpoints would provide further critical insight. Optimizing micronutrient supplementation, lifestyle interventions, and integrating genetic screening, such as MTHFR polymorphisms, with metabolic and epigenetic profiling may provide individualized strategies for managing HHcy in reproductive medicine. Collectively, these approaches hold promise for enhancing natural fertility, improving the outcome of assisted reproduction, and safeguarding the health of future generations.

Sample size, adjustment for confounders (e.g., age, diet, BMI, smoking) and ethnic/geographical stratification should be carefully considered as these factors can critically influence the association between Hcy metabolism and reproductive outcomes. Moreover, ethnic background, nutritional intake, supplementation, lifestyle, geographic variation, metabolic status, socioeconomic factors and pharmacological exposure all significantly influence the relationship between MTHFR polymorphisms, Hcy metabolism and fertility outcomes, emphasizing the necessity for future studies, incorporating well-matched controls and appropriate stratification, to ensure robust conclusions.

## Data Availability

Data sharing is not applicable to this article as no new data were created or analyzed in this study.

## Abbreviations

The following abbreviations are used in this manuscript:

BMI	Body mass index
CβS	Cystathionine β-synthase
Cys	Cysteine
DNA	Deoxyribonucleic acid
FF	Follicular fluid
FSH	Follicle-stimulating hormone
GnRH	Gonadotropin-releasing hormone
HHcy	Hyperhomocysteinemia
Hcy	Homocysteine
IVF	In vitro fertilization
MAT	Methionine adenosyltransferase
Met	Methionine
MS	Methionine synthase
MTHFR	5,10-Methylenetetrahydrofolate reductase
NO	Nitric oxide
PCOS	Polycystic ovary syndrome
RIF	Repeated implantation failure
RNA	Ribonucleic acid
SAH	S-adenosylhomocysteine
SAM	S-adenosylmethionine
SNP	Single nucleotide polymorphism
5-MTHF	5-methyltetrahydrofolate
THF	Tetrahydrofolate
